# Year-end reflections of *EVCNA* - 2022

**DOI:** 10.20517/evcna.2022.45

**Published:** 2022-12-30

**Authors:** Yoke Peng Loh

**Affiliations:** American Biochemist and Molecular Biologist, Bethesda, MD 20817, USA.

*Extracellular Vesicles and Circulating Nucleic Acids *(*EVCNA*) has completed another successful year in 2022, the 3rd year since its inception in December 2019. This year we have expanded our Editorial Board of eminent scientists to 46 members from 14 countries/regions with 7 Associate Editors. In addition, we now have an international junior Editorial Board of 59 young scientists who are supporting many aspects of the journal, including participating as reviewers and organizing Roundtable discussions and Academic Talks that we have added this year. In 2022, *EVCNA* published 30 papers with a total of 77,832 views, 14,527 downloads and 107 citations in Web of Science. Manuscript submissions were thoroughly peer-reviewed, and our rejection rate in 2022 was 45.5%. Currently, there are 13 Special Issues in progress covering diverse topics. They include Extracellular Vesicles in Intercellular Communication: A Decade of Achievements (Guest Editors: Randy Schekman and Y. Peng Loh), Extracellular Vesicles and miRNA in the Aging Process (Guest Editors: Consuelo Barras Blasco and Jorge Sanz-Rios), Circulating Nucleic Acids in Bodily Fluids: Noninvasive Prenatal Testing, Oncology, Transplant Monitoring and Beyond (Guest Editors: Eric Sistermans and Peiyong Jiang), Extracellular Vesicles and Neuroinflammation: Response to Infection, Injury and Aging Plus Vehicles for Treatment (Guest Editor: Lynn Pulliam), and Extracellular Vesicles in Tissue Remodelling in Health and Disease (Guest Editor: Sigrun Lange). All readers are welcome to submit to any of the Special Issues by contacting the Guest Editors.

The journal is indexed by Dimensions, CNKI Scholar and Google Scholar. Our articles funded by NIH and Europe PMC 37 funders are included in PMC. This year *EVCNA* became a member of COPE. *EVCNA* has several partners and sponsored meetings, including Research Gate, 3rd Liquid Biopsies Congress in Europe and the American Society of Intercellular Communications (ASIC) 2022 Annual Meeting.

Looking forward to 2023, the *EVCNA* editorial office is offering $600 each for two Best Paper Awards, a Review article and a Research article. We encourage you to submit your papers to *EVCNA*. Readers are invited to listen and participate in the upcoming Academic Talks and Roundtable Discussions announced on our website. If you are interested in organizing a Roundtable Discussion, please contact the Editorial Manager (managingeditor@evcna.com). We hope that scientists in the Extracellular Vesicle and Liquid Biopsy fields will continue to use *EVCNA* as a platform to share their work and opinions, commentaries and discussions with their fellow researchers. For 2023, the APC is still waived, which provides an opportunity for everyone to publish their experimental data or scholarly reviews and commentaries freely.

*EVCNA* would like to thank the Extracellular Vesicle and Liquid Biopsy research community for their great support of the journal in 2022 and look forward to their continued support and serving you in 2023.

Best Wishes to Everyone for Good Health and Success in 2023.

## DECLARATIONS

### Copyright

©The Author 2022.

